# Novel thermal stability enhanced xylanase improves the performance and digestibility parameters in broilers^☆^

**DOI:** 10.1016/j.psj.2024.104447

**Published:** 2024-10-28

**Authors:** Su Rin Lee, Daulat Rehman Khan, Jae Yong Park, Sebastian Kaczmarek, Eun Jung Choi

**Affiliations:** a Institute of Biotechnology, CJ CheilJedang Co., Suwon 16495, Republic of Korea; b CJ Europe GmbH, Unterschweinstiege 2-14 60549, Frankfurt am Main, Germany; c CJ Cheiljedang Co., Seoul 04560, Republic of Korea; d Department of Animal Nutrition, University of Poznan, Street Wołyńska 33 60-637 Poznan, Poland

**Keywords:** Broiler, Efficacy study, Stability, Xylanase, Xylanase inhibitor

## Abstract

Xylanases require thermal stability to withstand the pelleting process, pH stability to function in the gastrointestinal tract, and resistance to xylanase inhibitors in raw materials to be effective in animal feed. A GH11 family xylanase originating from an anaerobic fungus, *Orpinomyces* sp. strain PC-2, has high specific activity and resistance to xylanase inhibitors intrinsically. It was engineered using rational protein design methods to obtain a thermal and pH stable enzyme, OXynA-M. OXynA-M showed resistance to three types of xylanase inhibitors, *Triticum aestivum* xylanase inhibitors TAXI-IB and TAXI-IIA and xylanase inhibitor protein XIP and showed melting temperature of 87.2°C when measured using differential scanning calorimetry. It was stable at all pH between 2.0–10.0 incubated up to 4 h. Xylo-oligosaccharides (XOS) production profile using a wheat arabinoxylan substrate revealed the production of xylobioses up to xylohexaoses, which are known to have prebiotic functionalities. An animal trial was conducted in broiler chickens to evaluate the *in vivo* efficacy of the xylanase. In total, 600 1-day-old chickens were divided into six dietary treatments, including a positive control (PC) (T1) without the addition of exogenous enzyme and the rest where exogenous xylanase was added at the rates of 1200, 2400, 4800, 9600, and 240000 U/kg of feed from T2–T6. An increase in OXynA-M xylanase improved the performance parameters in the enzyme-treated groups compared with the control. The viscosity of ileal digesta decreased with increasing enzyme dosage. A significantly lower viscosity of 6.54 cP was determined for the minimum dose in T2 (1200 U/kg), and the viscosity was further reduced in T6 (240000 U/kg) (*P*<0.05) compared to the control treatment. The apparent ileal digestibility of crude protein, fat, and starch improved as the xylanase dosage increased. The application of OXynA-M xylanase improved the apparent ileal digestibility of crude protein when the dose was higher than that of T2 (1200 U/kg). Furthermore, the AME_n_ of the diets improved when xylanase was supplemented at a rate of 9600 U/kg (T5) compared with the control treatment (*P*<0.05).

## Introduction

A major portion of animal feed consists of plant and plant-based byproducts. Non-starch polysaccharides (NSPs) are abundantly present in the cell walls of plant-based raw materials, entrapping essential nutrients, such as protein and starch, which escape digestion in monogastric animals and are wasted. Despite various processing steps, such as toasting, grinding, and extraction, to release the entrapped nutrients, the intact cell wall remains in sufficient quantity in the finished feed. Exogenous NSP-degrading enzymes (NSPases), such as xylanase, are used in the commercial feed industry to maximize the release of entrapped nutrients. In addition, substrate degradation produces short-chain oligosaccharides that act as prebiotics for the gut microflora in monogastric animals (Ravn et al., 2017). Also, supplementation with exogenous xylanase reduces digesta viscosity and prevents vent pasting in poultry (Anwar et al., 2023; Barasch and Grimes, 2021).

Unfortunately, many natural enzymes have limitations that prevent their direct application in animal feed. They cannot endure high heat or extreme pH; thus, they need to be reinforced for thermal stability to withstand the pelleting process, pH stability to work while going through the gastrointestinal tract, and other functionalities, such as resistance to inhibitors in the case of xylanase, to be successfully applied for feed usage. We have developed a xylanase for feed application by initially selecting an enzyme with natural xylanase inhibitor resistance and then biotechnologically improving the enzyme for other characteristics.

Microbial pathogens invade plants by secreting a wide variety of cell wall degrading enzymes. These enzymes cleave the glycosidic bonds of plant cell wall polysaccharides, including cellulose and hemicellulose (Tundo et al., 2022). The major component of hemicellulose is β-1,4 linked xylan, thus consequently xylanases are used by phytopathogens to hydrolyze the xylan in the plant cell wall for invasion. Plants have evolved a defense mechanism in which protein inhibitors of xylanases, called xylanase inhibitors (XI), confer resistance against microbial xylanases, and thus against pathogen infection. However, these XIs also negatively affect exogenous xylanases, which are added to animal feed to improve the digestibility of raw materials. Even though XIs are proteins and the high-temperature pelleting process, a common practice in feed processing, decreases their inhibitory effects, residual inhibitors substantially decrease the activity of exogenous xylanases (Smeets et al., 2014). Thus, it is important to develop xylanases for feed application which can escape the inhibitory effects of XIs.

XIs are classified into different classes based on structure and specific inhibition properties. Major classes include xylanase inhibitor proteins (XIP) and *Triticum aestivum* xylanase inhibitors (TAXI), with different isoforms occurring within each class. XIs inhibit xylanases by binding to interface residues, forming protein-protein interactions that obstruct the active site and prevent xylan substrates from binding. Different XIs inhibit different families and origins of xylanases. Although some exceptions exist, in general, TAXI-type inhibitors inhibit glycoside hydrolase family 11 (GH11) xylanases and XIP-type inhibitors inhibit xylanases that originate from fungi and not from bacteria (Dornez et al., 2010; Gusakov, 2010). These characteristics make it possible to develop xylanases which are resistant to XIs by identifying xylanases which are naturally and intrinsically insensitive to XIs.

Using this strategy, we have developed a xylanase for feed application, starting with a GH11 family xylanase originating from *Orpinomyces* sp. strain PC-2 (OXynA). A GH11 xylanase was selected because of its high substrate specificity and large active site (Amerah et al., 2008). Xylanase originating from *Orpinomyces* was selected because of their possible resistance to XIs. *Orpinomyces* sp. strain PC-2 is a polycentric anaerobic fungus found in the rumen of herbivores (Ljungdahl, 2008). Rumen organisms, including anaerobic fungi, produce extensive amounts of diverse enzymes which are responsible for most of the plant cell wall degradation in the rumen. These hydrolytic enzymes show exceedingly high activities and analysis of these enzymes from rumen anaerobic fungi show high sequence similarity, similar guanine-cytosine (GC) content, and codon usage with bacterial enzymes rather than with aerobic fungi. They are also absent of introns, indicating the possibility of horizontal gene transfer from ruminal bacteria to ruminal anaerobic fungi (Garcia-Vallve et al., 2000). Based on this information, it was hypothesized that xylanase originating from *Orpinomyces* could have the XI insensitive characteristic of bacterial xylanases.

In many applications, including animal feed, high thermal and pH stability and the retention of high activity at the appropriate pH and temperature are crucial. In feed enzymes, thermal and pH stabilities can be acquired by applying coating to the enzyme. This method has been successful to varying degrees and is currently applied to many commercial feed enzymes. However, coating can have a negative impact on the availability of the enzyme after ingestion. It can delay the release of enzymes and cause reduction in the speed of activation (Amerah et al., 2019). Also coating the enzyme results in an additional increase in the production cost, which one prefers to avoid in commercial enzymes (Amerah et al., 2008). Therefore, instead of coating, modification of the protein sequence by protein engineering is commonly used to reinforce the intrinsic stability of feed enzymes. Such methods have been used to enhance the thermal and pH stability of OXynA.

To account for all the improved characteristics of the enzyme, an animal trial was conducted to determine the efficacy of OXynA-M, the engineered OXynA. Growth parameters, digesta viscosity, intestinal integrity parameters, and digestibility of major nutrients in wheat-based diet of fast-growing broiler chickens were evaluated.

## Materials and methods

### Strategy for the selection of *Orpinomyces* xylanase for engineering

To develop a xylanase for feed applications, xylanase originating from the rumen anaerobic fungus *Orpinomyces* sp. strain PC-2 was selected as the starting template for engineering. This selection was based on information from the literature, where 1) enzymes from rumen anaerobic fungi have high specific activity compared to enzymes from other sources (Ljungdahl, 2008), and 2) sequence and structural similarity was high to xylanase originating from another rumen anaerobic fungus *Neocallimastix patriciarum*, which is insensitive to XIs (Payan et al., 2004; Tundo et al., 2022). OXynA is composed of a single catalytic domain and two repeated peptide domains connected by a linker. Sequence identity between the catalytic domain of OXynA and each of the two catalytic domains of *Neocallimastix* xylanase is 89.9% and 88.7%, respectively (Li et al., 1997).

### Strains, vectors, and reagents

The pHCE plasmid (Takara, Japan) was used to clone the catalytic domain of OXynA. *E. coli* BL21(DE3) (Enzynomics, Korea) was used as the host for recombinant xylanase expression and library construction to select improved mutants. XIP1 (GenBank: AJ422119.1) and TAXI-IIA (GenBank: AJ697849.1) from *Triticum aestivum* were cloned into the pET21 vector (Takara, Japan) and expressed in BL21(DE3) cells. TAXI-IB (GenBank: AJ697851.1) was cloned into the pPICZaA vector (Invitrogen, Carlsbad, CA, USA) and expressed in *Pichia pastoris. Trichoderma reesei* derived from the RUTM strain and a Trichoderma expression vector derived from pT3C were used for the production of the enzyme (Li et al., 2007). Plasmid Miniprep and gel extraction kits were purchased from GeneAll Biotechnology (Korea). NiNTA resin was purchased from Qiagen (Germany) and beechwood xylan, from Megazyme Ltd. (Ireland). All other chemicals and reagents were purchased from Sigma-Aldrich.

### Structural modeling and rational design

**Fig. 1:**
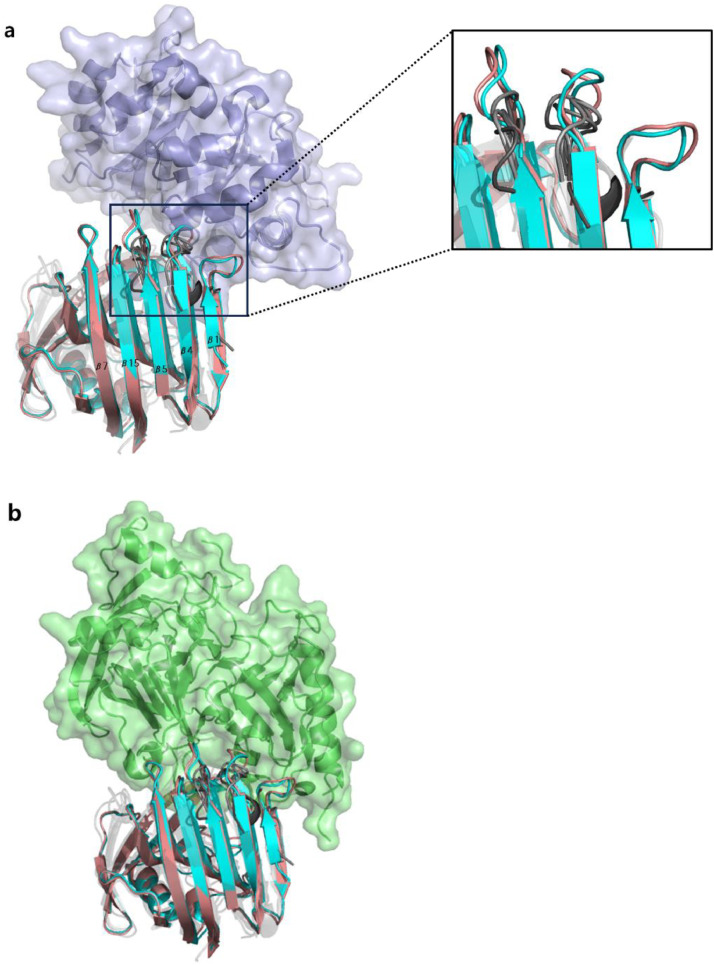
Three dimensional structures of xylanases from aerobic and anaerobic fungi with xylanase inhibitors XIP and TAXI. a The overlayed structure of OXynA model (light magenta), NpXynA (2C1F, cyan), *T reesei* XynII (1ENX, light gray), *T lanuguinosus* xylanase (1YNA, gray), *P funiculosus* XYNC (1TE1, dark gray), and *A nig*er xylanase I (1T6G, dim gray) is shown. All structures were superimposed onto the xylanase structure in 1TE1. The structure of XIP in the crystal structure 1TE1 is shown in purple in both cartoon and surface representation. All the loops of the aerobic fungi xylanases in different shades of gray are short and have similar structure to PfXYNC which interacts with XIP, whereas the loops of xylanases from the two anaerobic fungi, OXynA and NpXynA, are extended compared to the others and clash with the residues at the XIP interface. b The overlayed structures of all the xylanases superimposed to the xylanase structure in 1T6G is shown. The structure of TAXI in the crystal structure 1T6G is shown in green in both cartoon and surface representation. Similar to XIP, TAXI also clashes at the interface with the superimposed anaerobic xylanases.

Crystal structure of OXynA does not exist. A template-based structure-modeling protocol, GalaxyTBM (Ko et al., 2012) was used to construct a model structure of OXynA for structural analysis. Structural similarity between OXynA and *N. patriciarum* xylanase (NpXynA) was analyzed with OXynA model and NpXynA crystal structure (PDB ID 2C1F, (Vardakou et al., 2008)). OXynA model was superimposed to the crystal structure of *T. reesei* XynII bound to substrate, xylobiose and xylotriose (PDB ID 4HKW, (Wan et al., 2014)) to gather information about the residues that are close to the substrate. To prevent a decrease in activity when engineering the enzyme, amino acid residues participating in catalysis or situated inside the active-site cleft were not considered for mutagenesis. The OXynA model was also superimposed to the crystal structure of *Penicillium funiculosus* xylanase bound to XIP (PDB ID 1TE1, Fig. 1A, (Payan et al., 2004)) and *Aspergillus niger* xylanase bound to TAXI (PDB ID 1T6G, Fig. 1B, (Sansen et al., 2004)). This was used to gather information about the residues which interact with the xylanase inhibitors (Fig. 1). Residues at the interface were also not considered for mutagenesis. Disulfide bonds were selected based on Cα-Cα and Cβ-Cβ distance between the two residues in the OXynA model.

### Site-directed/site-saturation mutagenesis and protein expression/purification

Site-directed and site-saturation mutagenesis were performed using a QuickChange mutagenesis kit (Stratagene, USA). The plasmid pHCE containing the catalytic domain of the OXynA gene and a His6 tag at its C-terminus was used as a template to produce mutant xylanases, which were transformed into *E. coli* BL21(DE3) for recombinant expression.

For protein expression, the transformed BL21(DE3) cells were grown on LB medium. After the optical density at 600 nm reached 0.8, cells were collected by centrifugation and lysed by sonication. Proteins were attached to His6 tag Ni-affinity resin and purified using 50 mM sodium phosphate elution buffer with 250 mM imidazole adjusted to pH 8.0, with NaOH.

### Xylanase activity, thermal/pH stability and XI binding assay

Xylanase activity was determined using a spectrophotometric assay that detects the amount of reducing groups which react with dinitrosalicylic acid reagent (DNS). The assay was conducted in 5 ml reaction volumes containing 1.5 ml of 1% w/v beechwood xylan. The reaction was allowed to occur for 15 minutes at 50°C, pH 6.5 and terminated by the addition of 1.5 ml of DNS solution. The amount of reducing sugars released was measured at 540 nm. Under this reaction condition, one unit of xylanase activity is defined as the amount of enzyme required to release 1 μmol xylose per minute.

The thermal and pH stabilities were investigated by incubating the enzyme solution with 20 mM sodium phosphate buffer at various temperatures and pH values for different time periods. After pre-incubation, residual activity was determined using the results of the standard DNS activity assay described above. The activity value at the initial measurement under standard assay conditions was used as the control.

For the XI binding assay, the inhibitors purified using His6 tag Ni-affinity resin was diluted to 0.25 mg/ml or 0.5 mg/ml, mixed 1:1 with 0.1 mg/ml enzyme, and incubated at 37°C for 60 minutes. Further, 100 μl of 0.5% xylan from beechwood in 40 mM sodium phosphate buffer and 100 μl of diluted enzyme solution were reacted at 40°C for 15 minutes, and then the reaction was stopped by adding 300 μl of DNS solution. Color was developed by heating for 7 minutes at 100°C and after cooling, 500 μl of distilled water was added. The absorbance was measured at 550 nm.

### Xylo-oligosaccharide and Differential scanning calorimetry analysis

1 mg of 1% wheat arabinoxylan was treated with 1 U of diluted xylanase, allowed to react for 10, 20, 30, or 60 minutes, and then heated for 10 minutes to stop the reaction. The 5-fold diluted samples were analyzed using a Bio-LC system (ICS-3000; Thermo-Fisher, USA) equipped with a CarboPac PA1 column (4 × 250 mm) with an electrochemical detector (ED40). The sample was eluted with a 15%–100% linear gradient of 150 mM NaOH added to 600 mM sodium acetate at 1.0 ml/min flow rate.

Differential scanning calorimetry (DSC) was performed using a MicroCal PEAQ-DSC system (Malvern). All scans were run at pH 6.5 in 50 mM sodium phosphate buffer, in a temperature range from 20°C to 100°C at a rate of 90°C/h. The cell volume was 0.5 ml. Sodium phosphate buffer was used for baseline scans. The apparent melting temperature (Tm) value of xylanase was determined by analyzing the DSC scan data using MicroCal PEAQ-DSC software.

### Animal husbandry and dietary treatments

This study was conducted according to the European Union (EU) Directive 2010/63/EU for animal experiments at the Research Centre of the University of Poznan, Poland. The experiment was conducted with 600, 1-day-old male Ross 308 broilers in six treatments, with 10 replicates per treatment, and 10 birds per replicate. The total treatment duration was 35 days with a 3-phase feeding system: starter (1–10 d), grower (11–24 d), and finisher (25–35 d). The treatments were distributed as controls without the addition of exogenous xylanase and treatment groups supplemented with exogenous xylanase at 1200 (40 g/MT), 2400(80 g/MT), 4800(160 g/MT), and 9600 (320 g/MT) U/kg. The last treatment was supplemented with 240000 U/kg (8000 g/MT) to assess the tolerance of the supplemented enzyme in animals. The experimental diet was wheat and soybean meal based whereas, rye was used at 5% of the starter diets and 10% of the grower and finisher diets to induce high viscosity of digesta (Table 1). The analyzed xylanase activity values were consistent with the intended values.

**Table 1:** Ingredients and analyzed dietary composition of the experimental diets.

Ingredient (%)	0–10 d		11–24 d				25–35 d	
Wheat	56.03		49.56				52.17	
SBM	25.02		24.31				17.69	
Rye	5.00		10.00				10.00	
Sunflower meal	5.00		5.00				7.00	
Rapeseed oil	1.50		2.50				30	
Lard	1.68		2.59				3.44	
MCP	1.39		1.18				1.06	
Rapeseed meal	1.00		2.00				3.00	
Limestone	0.72		0.57				0.41	
L-Met	0.33		0.28				0.21	
NaCl	0.19		0.18				0.17	
L-Lys	0.37		0.29				0.31	
Thr	0.20		0.14				0.13	
L-Val	0.13		0.06				0.05	
L-Arg	0.14		0.07				0.05	
CJ Bio Xylanase*	0, 0.004, 0.008, 0.016, 0.032, and 0.80							
Nutrient composition %								
		Moisture	Crude fiber	Crude ash	Starch	Ca	P	Analyzed Xylanase Units/kg
0–10 d								
	T1	89.10	4.41	6.68	37.20	0.97	0.79	8.20
	T2	88.70	4.31	6.62	37.60	0.96	0.76	1353
	T3	90.60	4.37	6.47	36.80	0.96	0.77	2510
	T4	90.20	4.29	6.49	36.40	0.96	0.77	5346
	T5	90.10	4.50	6.71	36.10	0.95	0.76	10216
	T6	89.30	4.43	6.64	36.50	0.96	0.76	252932
11–24 d								
	T1	87.90	4.22	6.21	36.20	0.88	0.72	35.30
	T2	88.60	4.34	6.37	36.70	0.89	0.74	1284
	T3	88.40	4.32	6.31	36.60	0.88	0.74	3969
	T4	89.10	4.43	6.28	37.10	0.88	0.75	5855
	T5	89.00	4.29	6.32	37.20	0.89	0.75	13245
	T6	87.90	4.26	6.28	36.80	0.89	0.75	276443
25–35 d								
	T1	89.10	4.51	5.90	37.93	0.81	0.67	44.10
	T2	90.00	4.49	5.89	38.76	0.75	0.66	1234
	T3	89.80	4.54	5.98	38,5	0.79	0.67	2643
	T4	89.50	4.52	5.60	39.35	0.80	0.66	4954
	T5	90.20	4.50	5.87	39.43	0.80	0.68	10830
	T6	89.00	4.48	5.79	38.80	0.80	0.68	244190

⁎ : Conc. 36000U/g

The test feeds in mash form were prepared in a 100 kg capacity mixer for 4 minutes, with a mixing band of 27.4 rev/min. The finisher feed was supplemented with TiO_2_ (dose: 0.3%) as an indigestible marker to determine AME_n_ in finisher feed.

Performance parameters were determined for each feeding period and viscosity, nutrient digestibility, and intestinal integrity were determined at the end of the experiment (35 d). Mortality was recorded daily, whereas body weight and feed intake were recorded on day 0, coinciding with the change in feeding phase on day 10 and day 24 and at the end of the study on day 35. Feed conversion ratio (FCR) (mortality corrected) was calculated using body weight gain (BWG) and feed intake (FI).

To estimate AME_n_, excreta collection trays were installed in floor pens on day 28. After approximately 3 h, 12 samples were collected from each treatment group. The ileal contents (n = 12) were collected to estimate nutrient digestibility by the end of the experiment (35 d). The digesta were flushed from the terminal ileum (15 cm distal to the ileocecal junction), pooled in a cage (three birds per sample), freeze-dried, and ground, except for approximately 2 g of ileum content necessary for the viscosity test. A 1 cm sample of the lower part of the ileum was collected and stored at -80°C for later estimation of intestinal integrity parameters.

The experiment was conducted using a completely randomized design and growth performance data were analyzed by analysis of variance using the general linear model procedure of the R environment (R Development Core Team, 2014) and the “Agricolae” package (De Mendiburu, 2014). All data were presented as means with pooled standard errors. Mean separation was performed using Tukey's test (*P*<0.05).

### Laboratory analysis

Feed samples were analyzed in duplicate for crude protein (CP), crude fat (CF), gross energy (GE), calcium (Ca), NDF, and ADF using AOAC methods. Prior to analysis, excreta samples were homogenized using a stomacher homogenizer and freeze-dried. Ground excreta samples were analyzed for nitrogen content. The TiO_2_ content in the excreta and feed samples was determined according to the method described by Short et al. (1996). The gross energies of the excreta and feed samples were determined using an adiabatic bomb calorimeter (KL 12Mn; PRECYZJA-BIT PPHU, Bydgoszcz, Poland) standardized with benzoic acid. The AAs in the diets and digesta were determined using an AAA-400 Automatic Amino Acid Analyzer (Ingos Ltd., Prague, Czech Republic) with ninhydrin for post-column derivatization. Before analyses, the samples were hydrolyzed with 6 N HCl for 24 h at 110°C according to AOAC (2005) (procedure 994.12). Starch content was investigated using an agricultural diagnostic assay kit (Megazyme International) using thermostable α-amylase and amyloglucosidase according to AOAC (2005) (method 996.11).

To estimate ileal digesta viscosity, 2 g (wet weight) of fresh digesta was immediately placed in a microcentrifuge tube and centrifuged for 5 minutes. The supernatant was withdrawn and stored on ice until viscosity (mPa·s = cP = 1 × 100 dyne s cm^−2^) was determined using a Brookfield Digital DV-II+ cone/plate viscometer (Brookfield Engineering Laboratories Inc., Stoughton, MA, USA) at a shear rate of 42.5 sec^−1^ at 40°C. Viscosity was presented in centipoise units (cP) to compare the treatments. The quantitation of the endo-1,4-β-D-xylanase activity in feeds was performed using modified XylX6 assay (Megazyme, Wicklow, Ireland).

### AME and ileal digestibility

AME_n_ (kcal/kg) was calculated relative to the ratio of TiO_2_ to the content of the nutrient in question in the feed or excreta, according to the following equation:

AME_n_ = GE_diet_ − [GE_excreta_×(TiO_2,diet_/TiO_2,excreta_)] −8.22×{N_diet_ − [N_excreta_ ×(TiO_2,diet_/TiO_2,excreta_)}] where GE represents the gross energy [MJ/kg], N represents nitrogen, and TiO_2_ is a dietary marker. AME was corrected to a zero-nitrogen balance using 8.22 cal/kg N retained according to Hill and Anderson (1958). Ileal and total tract digestibility were calculated using the following equations (starch and digesta as an example):

### Digestibility = {1 − [(TiO_2,diet_/TiO_2,digesta_) × (Starch_digesta_/Starch_diet_)]}

where the contents of TiO_2_ and starch in the diets and digesta are expressed in grams per kilogram.

## Results

### Stability improvement of OXynA by rational design

For industrial applications, enzymes need to withstand extreme conditions. Common characteristics of feed enzymes are the capability to withstand 1) heat up to 85°C for 3 minutes in solution (J Ryu, CJ, personal communication) and 2) acidic pH of 2.5–3.5 which is the pH of the stomach of animals. Wildtype OXynA is known to retain 27% activity when incubated at 60°C for 30 minutes and almost no activity above 70°C (Trevizano et al., 2012).

We analyzed the modeled structure of OXynA and selected various residues that were considered to be designable (Fig. 1). The “designable residues” were those which are not involved in substrate binding or catalysis and would not affect the XI binding interface. An OXynA model structure superimposed to xylanase structures crystallized with a substrate or with xylanase inhibitors was used. Eight designable residues (G8, N28, N52, S66, S91, R105, Y162, and S178) were selected by structural analysis, and site-saturation mutagenesis was conducted on the eight sites using PCR. Out of the eight sites screened, mutations at two sites (28 and 52) showed 10–20 times higher residual activity at 70°C compared to the wildtype enzyme under the screening condition. To further increase the thermal stability, disulfide bonds were evaluated between any two residues with the correct configuration by mutating them to cysteine residues. Out of the many residue pairs which satisfied the appropriate geometric criteria (Cα-Cα distance between 4.0 Å and 7.0 Å and Cβ-Cβ distance of ≤ 5 Å, (Gao et al., 2020; Salam et al., 2014)), multiple designed disulfide bonds showed increase in residual activity at 70°C compared to the wildtype enzyme. Mutations from the saturation mutagenesis experiments and the disulfide design experiments were combined to form various combinatorial mutants of OXynA. Of these combinatorial mutants the best variant, named OXynA-M, composed of the following four mutations, R3C/N28A/T36C/N52P, showed over 2-fold increase in specific activity and retained over 90% residual activity when incubated for 10 minutes at 70°C compared to the wildtype OXynA.

### Characterization of OXynA-M heterologously expressed in *Trichoderma reesei*

*Trichoderma reesei* is a fungus which has a long history of being used for commercial enzyme production. It is known to secrete large amounts of protein and its production titer can go up as high as 80 g/L (Fonseca et al., 2020). The Trichoderma expression vector with OXynA-M insertion was transformed and enzyme production was confirmed by an activity assay of the culture supernatant. OXynA-M was purified from the supernatant and this heterologously expressed OXynA-M was used for functionality experiments (XIP assay, xylo-oligosaccharides (XOS) profile) and animal trials. The experimental methods described by Li et al. (2007) were followed for all steps in the production of OXynA-M in *Trichoderma*.

**Fig. 2:**
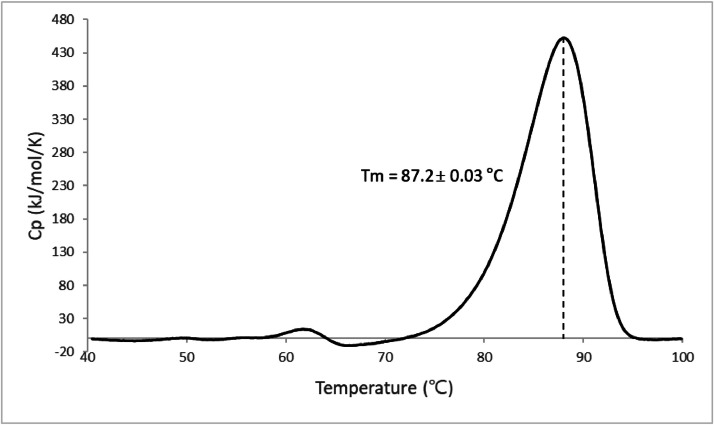
Differential scanning calorimetry result for OXynA-M. Three scans for 250 μg of OXynA-M in 0.5 ml 50 mM sodium phosphate buffer, pH 6.5 obtained at a scan rate of 1.5°C/min using a MicroCal PEAQ-DSC software. The apparent Tm value calculated using the DSC scan data was 87.2°C for OXynA-M.

To characterize the thermal stability of OXynA-M expressed in *Trichoderma*, we incubated the purified enzyme for 3 minutes at 80°C, 85°C, and 90°C. OXynA-M retained over 90%, 80%, and 53% of residual activity under these conditions, respectively. Residual activity of over 80% when incubated at 85°C for 3 minutes condition allows for feed application without the need for coating or other methods to reinforce thermal stability. Thermal stability was also evaluated using differential scanning calorimetry. The Tm of purified OXynA-M was 87.2°C, significantly higher than many feed xylanases in the market (Fig. 2). The pH stability was also checked for this thermal stable enzyme, and it was observed that activity was retained even after 4 h of incubation at all pH ranges between 2.0–10.0.

### XIP insensitivity study of the thermal stable mutant OXynA-M

**Fig. 3:**
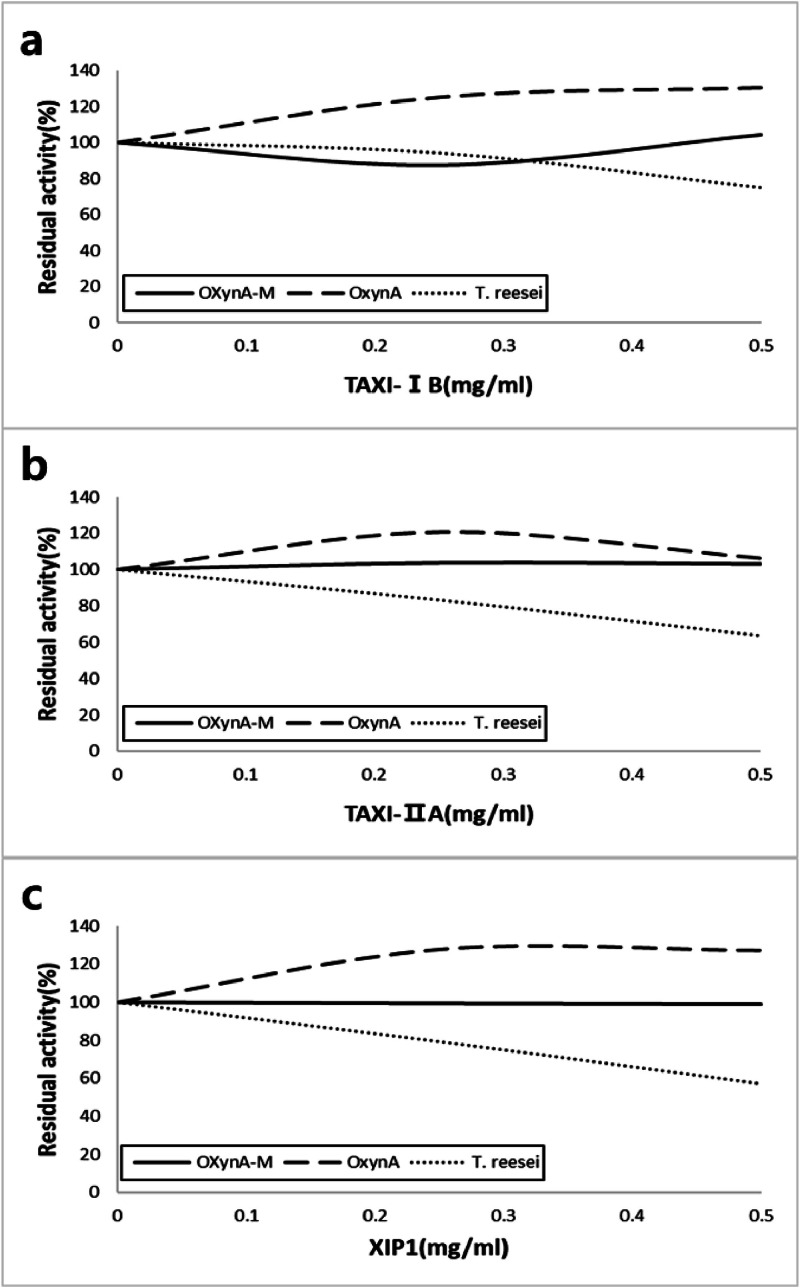
XI binding assay of OXynA-M, OXynA, and XynII from *T. reesei*. XIs were added to each of the enzymes and incubated at 37°C for 60 minutes. Residual activities were measured after incubation. a TAXI-IB inhibition graph, b TAXI-IIA inhibition graph, and c XIP inhibition graph. As predicted, OXynA was uninhibited by all three XIs as was our engineered xylanase OXynA-M. On the other hand, XynII from *T. reesei*, known to be inhibited by both XIP and TAXI class of inhibitors, showed decreasing residual activity with increasing amount of each of the XIs.

OXynA-M was engineered from OXynA, which was predicted to have resistance to XI inhibition. However, its insensitivity to XIs needed to be confirmed. Two isomers of TAXI, TAXI-IB and TAXI-IIA and XIP-I from *Triticum aestivum* were recombinantly expressed in *E. coli* or *P. pastoris* and purified for use in xylanase inhibition assays. Fig. 3 shows the residual activity of OXynA wildtype, thermal-pH stable variant OXynA-M, and XynII from *T. reesei* (TrXynII), an aerobic fungus xylanase known to be inhibited by XIs, after being mixed and incubated for 1 h with different amounts of each of the three types of inhibitors. As predicted OXynA showed resistance to inhibition from both TAXI and XIP class. Engineering of OXynA to a thermal and pH-stable variant, OXynA-M, retained this feature. TrXynII on the other hand was inhibited by all three XIs as referenced in the literature (Tundo et al., 2022).

### Xylo-oligosaccharide hydrolysis pattern analysis

**Fig. 4:**
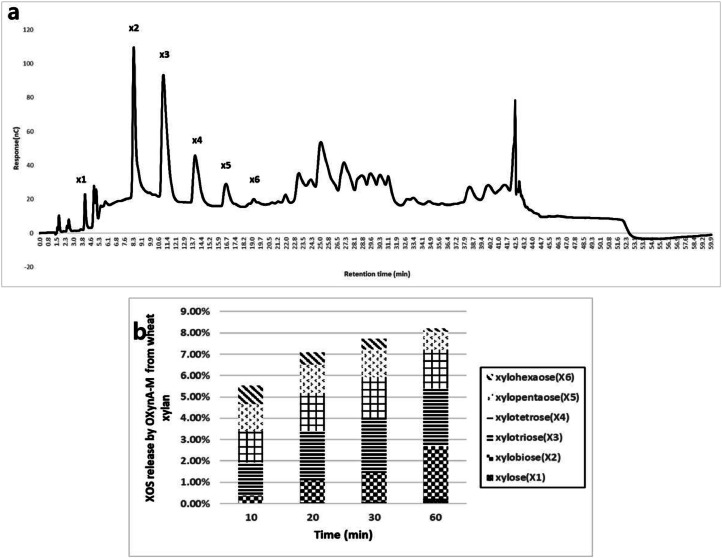
XOS production of OXynA-M. a A representative HPLC chromatogram of XOS produced by OXynA-M from wheat arabinoxylan substrate incubated for 30 minutes. X1: xylose, X2: xylobiose, X4: xylotriose, X4: xylotetrose, X5: xylopentaose, and X6: xylohexaose. b Xylobiose to xylopentaose, known for their prebiotic effect on beneficial gut microbials, such as *Bifidobacterium* and *Lactobacillus*, was produced in a notable amount by OXynA-M when arabinoxylan from wheat was used as substrate.

OXynA-M was examined to see if XOS with low DP of 2 to 6, which are known to have prebiotic effects and are beneficial to gut health, are produced (Gullón et al., 2008; Huang et al., 2019). XOS production by enzymatic hydrolysis was measured by varying the reaction time. When wheat arabinoxylan was used as the substrate, OXynA-M produced notable amounts of functional XOS, xylobiose up to xylopentaose, and only trace amounts of xylose monomers (Fig. 4).

### Performance and ileal digesta viscosity

A gradual increase in exogenous xylanase improved the performance parameters in the enzyme supplementation treatments compared with the control. During the starter period, a significant (*P*<0.05) improvement was observed in BWG at the lowest dose of 1200 U/kg in T2 compared to that in T1 (control). The difference in BWG at the starter stage was not carried over to the grower or finisher periods. No significant difference was observed in any of the treatments except T6 (240000 U/kg), the tolerance group, which showed the highest BWG (2265 g) during the entire study period (*P*<0.05) from 0–35 d (Table 2).

**Table 2:** Performance parameters, ileal digestibility, and viscosity in broilers fed with control and xylanase supplemented diets from 0 to 35th d.

Parameters	Treatments								
	Period	T1 Control	T2 1200 U/kg	T3 2400 U/kg	T4 4800 U/kg	T5 9600 U/kg	T6 240000 U/kg	SEM	P
Body weight gain (g)									
	0–10 d	231^b^	247^a^	251^a^	248^a^	250^a^	249^a^	1.572	<0.001
	11–24 d	867b	895^ab^	902^ab^	919^ab^	917^ab^	940^a^	6.042	0.009
	25–35 d	1010	1043	1017	1053	1051	1076	8.636	0.241
	0–35 d	2107^c^	2185^abc^	2170^bc^	2219^ab^	2218^ab^	2265^a^	12.986	0.009
Feed Intake (g)									
	0–10 d	247	251	255	252	249	252	1.960	0.9199
	11–24 d	1207	1219	1191	1231	1224	1215	7.565	0.7366
	25–35 d	1676	1688	1674	1678	1692	1684	10.704	0.997
	0–35 d	3130	3158	3120	3162	3165	3152	15.723	0.956
FCR (g/g)									
	0–10 d	1.069^a^	1.015^b^	1.015^b^	1.017^b^	0.998^b^	1,015	0.006	0.041
	11–24 d	1.393^a^	1.365^ab^	1.321^bc^	1.341^abc^	1.335^bc^	1,294^c^	0.007	0.005
	25–35 d	1.637	1.619	1.648	1.597	1.612	1.565	0.010	0.084
	0–35 d	1.486^a^	1.445^b^	1.439^b^	1.425^bc^	1.428^bc^	1.392^c^	0.005	<0.001
Ileal digest viscosity (cPs)									
	35 d	10.10^a^	6.54^b^	4.65^bc^	3.36^c^	2.75^c^	2.83^c^	0.4	<0.001

abc = *P*<0.05, SEM – pooled standard error of the mean

In the starter period (0–10 d), the feed conversion ratio improved (*P*<0.05) in T2 (1200 U/kg) compared to that in the control treatment (T1). In the later periods, growers and finishers, all treatments showed similar FCR except for the tolerance (T6:240000 U/kg) treatment, which showed an improved FCR (1.392) compared to the control T1 (1.486) (*P*<0.05).

Considering the entire study period (0–35 d), T2 (1200U/kg) showed a significant (*P*<0.05) improvement in FCR (1.445) compared to T1 (1.486). However, no difference was observed in T3–T5 (2400–9600 U/kg), whereas T6 (240000U/kg) revealed a significant (*P*<0.05) improvement in FCR (1.392) when compared to T1–T5 (1.486, 1.445, 1.439, 1.425 and 1.428) (Table 2).

### Ileal digest viscosity

Digesta viscosity was measured at the end of the study period and a gradual decrease was observed in digesta viscosity with a gradual increase in xylanase dosage. A statistically significant (*P*<0.05) improvement was observed at the first enzyme dose of 1200 U/kg (T2) compared to the control treatment (T1). The next significant (*P*<0.05) improvement in digesta viscosity (3.36, 2.75, and 2.83 cPs) was observed in T4–T6 (4800-240000 U/kg) compared to T3–T1 (4.65, 6.54, and 10.10 cPs) (2400-0 U/kg) (Table 2).

### Ileal digestibility and AME_n_

The apparent ileal digestibility of crude protein, fat, and starch increased with increasing xylanase dose. Xylanase improved ileal CP digestibility at doses higher than 1200 U/kg (T2). In the case of crude fat ileal digestibility, all dosages effectively increased in comparison with the control treatment (*P*<0.05). Ileal starch digestibility improved when xylanase was used at 2400 U/kg or higher (>T3).

Furthermore, the AME_n_ of the diets improved when xylanase was used at a 4x dose (T4:4800 U/kg) compared to the control treatment (T1) (*P*<0.05). Details of the apparent digestibility and AME_n_ are presented for major nutrients (Table 3).

**Table 3:** Apparent ileal digestibility (in %) of crude protein, crude fat, starch or AME_n_ of diets in broilers fed with control and xylanase supplemented diets at 35th d.

	Apparent ileal digestibility (in %) of			AME_n_
	Crude protein	Crude fat	Starch	
Treatments				
T1 (Control)	69.50^d^	57.60^b^	85.60^c^	2684^b^
T2 (1200U/kg)	73.30^b^	70.20^a^	87.80^bc^	2766^b^
T3 (2400U/kg)	74.30^bc^	69.20^a^	91.50^ab^	2814^b^
T4 (4800U/kg)	77.30^ac^	74.20^a^	91.40^ab^	2922^ab^
T5 (9600U/kg)	78.10^a^	71.40^a^	93.00^a^	3054^a^
T6 (240000U/kg)	79.70^a^	73.70^a^	93.80^a^	3139^a^
				
SEM	0.624	1.331	0.634	37.440
P	<0.001	0.0016	<0.001	0.001

abc = *P*<0.05, SEM – pooled standard error of the mean

### Intestinal integrity parameters

Intestinal samples were collected at the end of the experimental period and no significant (*P*>0.05) differences were observed among the enzyme treatments compared to the control (Table 4).

**Table 4:** Protein carbonyl, MDA, IL-8, TNF, IgG, and IgA in broilers fed with control and xylanase supplemented diets on the 35th d.

Treatments	Protein carbonyl [nmol/mg protein]	MDA [nmol/mg of proteins]	IL-8 [ng/mg of protein)	TNF alpha [pg/mg of protein]	IgG [ng/mg of protein]	IgA [ng/mg of protein]
T1 (Control)	2.916	1.521	129.70	27.90	127.40	213.60
T2 (1200U/kg)	1.992	1.160	110.90	27.50	134.90	228.20
T3 (2400U/kg)	2.567	1.246	118.80	28.70	149.30	209.90
T4 (4800U/kg)	1.930	1.401	130.60	26.90	130.30	238.80
T5 (9600U/kg)	1.682	1.032	119.40	28.80	117.40	173.40
T6 (240000U/kg)	2.215	1.089	117.40	28.10	133.10	194.30
						
SEM	0.171	0.086	2.956	0.255	3.365	9.502
P	0.331	0.572	0.360	0.250	0.148	0.417

SEM – pooled standard error of the mean

abc = *P*<0.05, SEM – pooled standard error of the mean

## Discussion

Various attempts have been made to eliminate sensitivity of xylanases to XI in feed applications. Appropriate residues for site-directed mutagenesis at the interface between xylanase and XI were selected to abolish the interaction and allow resistance of xylanase to XIs. Sorensen and Sibbesen (2006) mutated *Bacillus subtilis* xylanase A, in which multiple residues outside the active site were selected by structural evaluation of TAXI bound to the xylanase. These residues were mutated to other amino acids using site-directed mutagenesis and screened for variants that were not inhibited by TAXI, resulting in the acquisition of three mutants that were completely insensitive to TAXI. However, the mutagenesis of residues at the interface often results in decreased activity, which prevents the engineered enzyme from being used for industrial purposes (Rasmussen et al., 2010; Tahir et al., 2004).

We selected a different approach where a xylanase with intrinsic resistance to XIs was developed as feed xylanase. A GH11 xylanase originating from a ruminal anaerobic fungus *Orpinomyces* sp. strain PC-2 was assumed to be insensitive to XIs due to the possibility of horizontal gene transfer of hydrolytic enzymes from bacteria to anaerobic fungi in the rumen. From the high structural and sequential similarity (Li et al., 1997) to *Neocallimastix patriciarum* xylanase (NpXynA), another anaerobic rumen fungus originating xylanase already known to be insensitive to xylanase inhibitors XIP and TAXI (Payan et al., 2004; Tundo et al., 2022), this prediction was reinforced. Confirmation of the structural similarity between the two enzymes was conducted by comparing the crystal structure of NpXynA and model structure of OXynA. Both structures show the characteristic β jelly-roll fold comprised of two β sheets of the GH11 family. Antiparallel β strands which compose the two sheets bend to produce the substrate binding groove of the active site. The β sheet in direct contact with the substrate is comprised of nine β strands in the following order, β2, β3, β6, β14, β8, β9, β12, β11, and β10. Comparison of the crystal structures of GH11 family of xylanases from *Trichoderma reesei* (PDB ID 1ENX, (Torronen and Rouvinen, 1995)), *Penicillium funiculosus* (PDB ID 1TE1, (Payan et al., 2004)), *Thermomyces lanuginosus* (PDB ID 1YNA, (Gruber, et al., 1998)), and *Aspergillus niger* (PDB ID 1T6G, (Sansen et al., 2004)), all aerobic fungi xylanases known to be sensitive to xylanase inhibitor XIP, showed significant structural difference with the anaerobic fungi originating xylanases OXynA and NpXynA in the loop region connecting the β strands, especially the loop between β1/β2, β3/β4, and β5/β6 (Fig. 1A). These loops are longer than their aerobic xylanase counterparts and will structurally clash with the XIP when the two proteins come together, as can be inferred from the superimposed structures of the 1TE1 and OXynA model (Fig. 1A). These loops also appear to clash with TAXI when 1T6G and OXynA are superimposed (Fig. 1B). Additionally, in the case of TAXI, the loop between β7/β8 also seems to be involved in the interaction between xylanases and the XI. The longer loop between β7/β8 of the xylanases from the anaerobic fungi compared to the aerobic fungi-originated xylanases might also contribute to the insensitivity to TAXI. Based on this structural characteristic of OXynA and its high sequence similarity to NpXynA, we predicted that OXynA will be insensitive to XIP and TAXI. This was experimentally confirmed by the XI assay results shown in Fig. 3. Structural analysis comparing xylanases originating from aerobic fungi with OXynA and NpXynA provided structural insights into how xylanases originating from anaerobic fungi do not bind to XIs. However, this speculation needs to be validated by mutational experiments and more detailed structural analyses.

High activity was another key reason for the selection of OXynA as the starting template for engineering. Most hydrolytic enzymes of anaerobic fungi have higher specific activities than those of their aerobic counterparts. The native XynA of *Orpinomyces* sp. strain PC-2 s reported to have a specific activity of 3500–4500 U/mg against beechwood xylan compared to xylanases from other sources ranging from 20 to 600 U/mg (Li et al., 2007; Ljungdahl, 2008; Trevizano et al., 2012). Xylanase A from *Neocallimastix patricium* also has a comparable high specific activity of 6000 U/mg against oat spelt xylan (Gilbert et al., 1992). In our analysis, OXynA showed a specific activity of 2535±28 U/mg and the engineered OXynA-M showed specific activity of 6548±864 U/mg when using beechwood xylan, showing an increase of over 2-fold compared to the wildtype OXynA.

There are various methods of protein engineering including experimental methods, such as directed evolution and rational methods, such as computational protein design. These approaches have been used to modify the functionality of many different proteins used in industry, including stability, catalytic efficiency, and substrate specificity (Choi et al., 2009; Liu et al., 2019; Victorino da Silva Amatto et al., 2021). Xylanases that have been engineered for stability can be found in vast amount of literatures (Pandey et al., 2023). In the present study, the thermal and pH stabilities of OXynA were engineered using various experimental and rational design methods. Combination of various improved mutants resulted in an extremely thermal and acid-stable xylanase that retained resistance to XIs. This enzyme, OXynA-M, is now used commercially as a feed additive, sold as CJ Xylanase (CJ Youtell, Shandong, China). In this engineered enzyme, all mutations occur far away from the active site, and thus, the activity of each is either retained or increased with over 2-fold overall increase in specific activity. The thermal stability of the enzyme is synergistically increased with each mutation. The improved OXynA-M was assessed for the necessary biochemical characteristics (activity, thermal stability, and pH stability) and showed extremely enhanced results. The functionalities required by feed enzymes were also evaluated (XI inhibition and XOS production profiles). Inhibition by two major classes of XIs were not detected. Xylo-oligosaccharide is an emerging prebiotic for animal health and nutrition (Moura et al., 2007). XOS are highly resistant to gastrointestinal enzymes and gastric acids, which allows them to pass through the upper gastrointestinal tract and reach the lower intestine. There they are metabolized by beneficial microbes, such as *Bifidobacterium* and *Lactobacillus,* into short-chain fatty acids, which are beneficial to the host because of their immunomodulatory, anticarcinogenic, antimicrobial, and antioxidant properties (Mano et al., 2018; Samanta et al., 2015). Xylobiose up to xylotetrose are suggested to be the preferred substrate for *Bifidobacterium* and *Lactobacillus* (Gullón et al., 2008; Moura et al., 2007) and OXynA-M produces a notable amount of xylobiose up to xylopentaose during 1hr incubation. In addition, OXynA-M produced only trace amounts of xylose, which does not have prebiotic effects and can be detrimental to animals in large amounts (Fig. 4).

The outstanding *in vitro* characteristics (high thermal and pH stability, high activity, XI resistance, and low DP XOS production) observed for OXynA-M needed to be confirmed *in vivo*. The underlying mechanism of xylanase supplementation in feed utilization and consequent animal growth is complex and it will be difficult to contribute the effect of each improved characteristic of OXynA-M to *in vivo* performance. However, the negative correlation between xylanase inhibition level on growth rate has been observed in broiler experiments (Krogh Madsen et al., 2018). The amount of XI levels differs between the types of cereals, with the highest level of inhibition observed in wheat and rye, and extremely low or null in barley, maize, oat, and rice (Goesaert et al., 2004). Based on this information, we expect the best performance of our XI-resistant xylanase in a wheat-based diet compared to other cereal-based feeds. Hereby, a broiler trial test was conducted for OXynA-M using a rye-added wheat-based diet.

The improvement observed in the performance parameters with xylanase supplementation has been attributed to the release of entrapped nutrients from the cell walls of the vegetable raw materials. Ravn et al. (2016) reported the effect under the microscopic visualization on wheat, barley and rye treated by GH11 xylanase. They demonstrated solubilization of cell walls by the application of xylanase. In addition, the application of GH11 xylanases significantly improved the performance parameters (*P*<0.05), especially BWG and FCR in the starter period (0–10 d) and FCR during the entire study period. No difference in feed intake was observed among the treatments. Similar findings were observed by Luo et al. (2009), where they supplemented plants with xylanase at doses of 0, 500, 1000, and 5000 U/kg. Supplementation with xylanase significantly improved the feed conversion ratio (FCR) during days 1–21 and days 1–42 (*P*<0.05); however, no notable improvement was observed in body weight gain (BWG). In a related study by Gorenz et al. (2022), xylanase was added to the diet at levels of 30000 U/MT, 45000 U/MT, and 90000 U/MT without any performance differences, even though the energy content of the negative control (NC) diet decreased by 125 Kcal ME/kg. When xylanase was supplemented at 15 or 30 g/ton in the NC diet, a significant improvement (*P*<0.05) in body weight gain was observed compared with the positive control (PC). The 30 g/ton dosage also resulted in feed intake levels that matched those of the PC. Regardless of the amount used, xylanase supplementation improved the FCR (*P*<0.05) compared to the NC, effectively aligning the results with those of the PC. Several other studies have demonstrated the positive effect on the performance of broilers when supplemented with exogenous xylanase (Arczewska-Wlosek et al., 2019; Van Hoeck et al., 2021; Vasanthakumari et al., 2023; Zhang et al., 2014).

In the present study, the digesta contents were collected at the end of the experiment and the viscosity was measured. The digesta viscosity was reduced significantly at the minimum dose of xylanase of 1200U/kg (T2) compared to that of the control (T1). Similarly, earlier studies have demonstrated the effect of exogenous xylanase on the reduction in digesta viscosity in broilers. Van Hoeck et al. (2021) found that the inclusion of xylanase in broiler diets significantly reduced ileal viscosity, leading to improved nutrient digestibility and growth performance. Similarly, Barasch and Grimes (2021) reported that xylanase supplementation effectively decreased the ileal digesta viscosity in broilers, contributing to enhanced nutrient utilization and overall performance.

Of the ileal digesta collected at the end of the study period, crude protein, fat, and starch digestibility improved significantly at the lowest dose of xylanase (T2:1200 U/kg) compared to the control (T1). This effect was further enhanced at higher doses of xylanase in T5–T6 (9600–240000 U/kg) than at 1200 U/kg (T2) and 2400 U/kg (T3), with statistical significance (*P*<0.05). AME_n_ did not improve in T2 (1200 U/kg) and T3 (2400 U/kg) compared to T1; however, the difference became significant in T5 (9600 U/kg) onward compared to T1–T4 (0–4800 U/kg). This can be attributed to the higher doses of xylanase in T5 (9600 U/kg) and T6 (240000 U/kg). A higher dosage reduced digesta viscosity even further than T1–T4 (0–4800 U/kg), and a lower digesta viscosity potentially lead to an increase in nutrient digestibility and improved AME_n_ (Vasanthakumari et al., 2023).

Intestinal integrity parameters, including protein carbonyl, MDA, IL-8, TNF, IgG, and IgA, were analyzed in the intestinal samples at 35 d. Exogenous xylanase supplementation did not result in any statistically significant (*P*<0.05) difference between the treatments and control. Based on the current investigations by the authors, the literature on the antioxidant capacity of the intestine following supplementation with exogenous xylanase is limited. Wang et al. (2021) conducted the experiment along with the performance parameters, they measured intestinal microbiota composition and few of the antioxidant capacity parameters including, interleukin-6, tumor necrosis factor-α, and malondialdehyde content in broilers, and concluded the positive effects on the intestinal health of young broilers. However, this effect was not statistically significant (*P*<0.05). Similar results were observed in the present study.

## Conclusion

A GH11 family xylanase originating from *Orpinomyces* sp. strain PC-2 was used to develop a xylanase that is well suited for feed applications. Its pH and thermal stability were improved by protein engineering methods and the Tm of the engineered xylanase was 87.2°C measured by DSC. The specific activity of the enzyme increased over 2-fold, and it retained the XI-resistant property of the original enzyme. The XOS production profile showed a high production of xylobiose to xylopentaose, which is known to act as a prebiotic in animals. These outstanding in vitro characteristics were confirmed in an animal trial using a wheat-based diet, in which the XI resistance of the engineered xylanase is assumed to have the greatest positive effect. The performance and digestibility parameters of broiler chickens improved significantly when the engineered xylanase was supplemented.

## List of abbreviations

AA: Amino acid

ADF: Acid detergent fiber fraction

AME_n_: Apparent metabolizable energy, N-corrected

BWG: Body weight gain

CP: Crude protein

CF: Crude fat

DNS: Dinitrosalicylic acid

DP: Degree of polymerization

DSC: Differential scanning calorimetry

FCR: Feed conversion ratio

FI: Feed intake

GC: Guanosine-cytosine

GE: Gross energy

GH11: Glycoside hydrolase family 11

IgA: Immunoglobulin A

IgG: Immunoglobulin G

IL-8: Interleukin-8

MDA: Malondialdehyde

NaOH: Sodium hydroxide

NDF: Neutral detergent fiber fraction

NiNTA: Nickel nitrilotriacetic acid

NSP: Nonstarch polysaccharide

TAXI: *Triticum aestivum* xylanase inhibitor

TiO_2_: Titanium dioxide

TNF-α: Tumor necrosis factor - alpha

XI: Xylanase inhibitor

XIP: Xylanase inhibitor protein

XOS: Xylo-oligosaccharide

## Funding

This study was funded by CJ CheilJedang (Seoul, Korea).

## Authors’ contributions

Conceptualization and design, EJC, DRK, JYP; methodology, EJC, SK, SRL; conduction of experiment, SRL, SK; data analysis, EJC, SK, SRL; data interpretation EJC, SRL, DRK, SK; manuscript preparation EJC, DRK, SRL.

All authors have read and agreed to the final version of the manuscript.

## Declaration of competing interests

The authors declare the following financial interests/personal relationships which may be considered as potential competing interests: Eun Jung Choi reports a relationship with CJ CheilJedang Corp that includes: employment. Lee Su Rin reports a relationship with CJ CheilJedang Corp that includes: employment. Park Jae Yong reports a relationship with CJ CheilJedang Corp that includes: employment. Daulat Rehman Khan reports a relationship with CJ CheilJedang Corp that includes: employment. Eun Jung Choi has patent issued to Cheiljedang. Sebastian A. Kaczmarek, PhD(Assoc. Professor of Poznan University of Life Sciences, Poznan, Poland) is associated editor of the journal If there are other authors, they declare that they have no known competing financial interests or personal relationships that could have appeared to influence the work reported in this paper.

## Data Availability

All data generated or analyzed during this study are available from the corresponding author upon reasonable request.
